# The role of neutrophils in bronchiectasis

**DOI:** 10.1080/07853890.2025.2584413

**Published:** 2025-11-06

**Authors:** Jifang Liang, Xueli Bai, Xiansheng Liu

**Affiliations:** Department of Pulmonary and Critical Care Medicine, Third Hospital of Shanxi Medical University, Shanxi Bethune Hospital, Shanxi Academy of Medical Sciences, Tongji Shanxi Hospital, Taiyuan, China

**Keywords:** Bronchiectasis, bronchodilation, neutrophils, inflammatory mechanisms, activation inhibition, targeted therapy

## Abstract

**Background:**

Neutrophils are pivotal inflammatory cells in bronchiectasis pathophysiology, yet their stage-specific roles remain incompletely understood. This review synthesizes evidence on neutrophil activation across disease stages and explores therapeutic implications.

**Methods:**

We performed a thorough literature review analyzing neutrophil behavior in bronchiectasis, focusing on proliferation, activation, and their contributions to tissue damage during the early and middle stages and analyzing their behavior and its correlation with disease progression. To ensure a comprehensive review of the literature on the role of neutrophils in bronchiectasis, we conducted a systematic search using the following databases: PubMed, Embase, and Web of Science. The search terms included ‘neutrophils,’ ‘bronchiectasis,’ ‘neutrophil elastase,’ ‘bronchiectasis treatment,’ and ‘neutrophilic inflammation’. The search period covered articles published from January 2000 to June 2024. We also reviewed the reference lists of relevant articles to identify additional studies.

**Results:**

Neutrophils demonstrated significant proliferation and activation during the early and middle stages of bronchiectasis, leading to the release of inflammatory mediators and an exacerbation of tissue damage. In particular, neutrophil activation during the middle stage of the disease was significantly positively correlated with the destruction of bronchial tissue. Furthermore, inhibiting neutrophil activation markedly reduced the release of inflammatory factors and improved the integrity of bronchial epithelial cells.

**Conclusions:**

This study highlights the role of neutrophil activation at different stages of bronchiectasis and suggests that targeting neutrophil activation may represent a promising therapeutic strategy.

Bronchiectasis is a prevalent chronic airway inflammatory disease, ranking just behind chronic obstructive pulmonary disease and asthma. It is characterized by various etiologies leading to bronchial tree disease, resulting in rational and permanent dilation, as well as recurrent suppurative infections. The clinical manifestations of bronchiectasis include a chronic cough, the expectoration of large volumes of purulent sputum, and, in some cases, hemoptysis. Severe instances can lead to significant damage to lung tissue and function, adversely affecting patients’ quality of life [1]. A survey conducted on the prevalence of bronchiectasis in the United States from 2000 to 2015 revealed that the prevalence among Asian individuals is significantly higher compared to white and black individuals, suggesting a greater susceptibility to bronchiectasis in the Asian population [2]. While bronchiectasis is not uncommon in China, it has not garnered sufficient attention, and there remains a lack of large-scale epidemiological data. The recurrent acute exacerbations of bronchiectasis impose a substantial social and economic burden, rendering it a global public health concern. In patients with bronchiectasis, chronic inflammatory reactions in the airways predominantly involve the aggregation of neutrophils and the secondary secretion of various inflammatory mediators. Following the activation of numerous neutrophils, mediators such as proteases, reactive oxygen species, and cellular factors are released, leading to a significant increase in the levels of inflammatory mediators in the sputum of these patients [3]. This study aims to review the role of neutrophils in bronchiectasis, providing a feasible framework for the diagnosis and treatment of this condition.

## 1. Understanding of neutrophils in bronchiectasis

Bronchiectasis is a chronic respiratory disease characterized by irreversible dilation of the bronchi. It is frequently associated with chronic cough, excessive sputum production, and recurrent respiratory infections. The etiology of bronchiectasis is multifactorial, potentially arising from infections, immune deficiencies, genetic predispositions, or other underlying diseases, which contribute to structural destruction and dysfunction of the bronchial wall [4]. The pathogenesis of this disease is complex and involves the interplay of various cells and molecules. Pathological features of bronchiectasis encompass inflammation and fibrosis of the bronchial wall, dilation and deformation of the bronchial lumens, as well as damage to and loss of function of the cilia [5]. These pathological changes result in a decline in airway clearance function, thereby increasing the risk of infection. Persistent infection and inflammation further exacerbate bronchial damage, perpetuating a vicious cycle.

**Figure 1.**
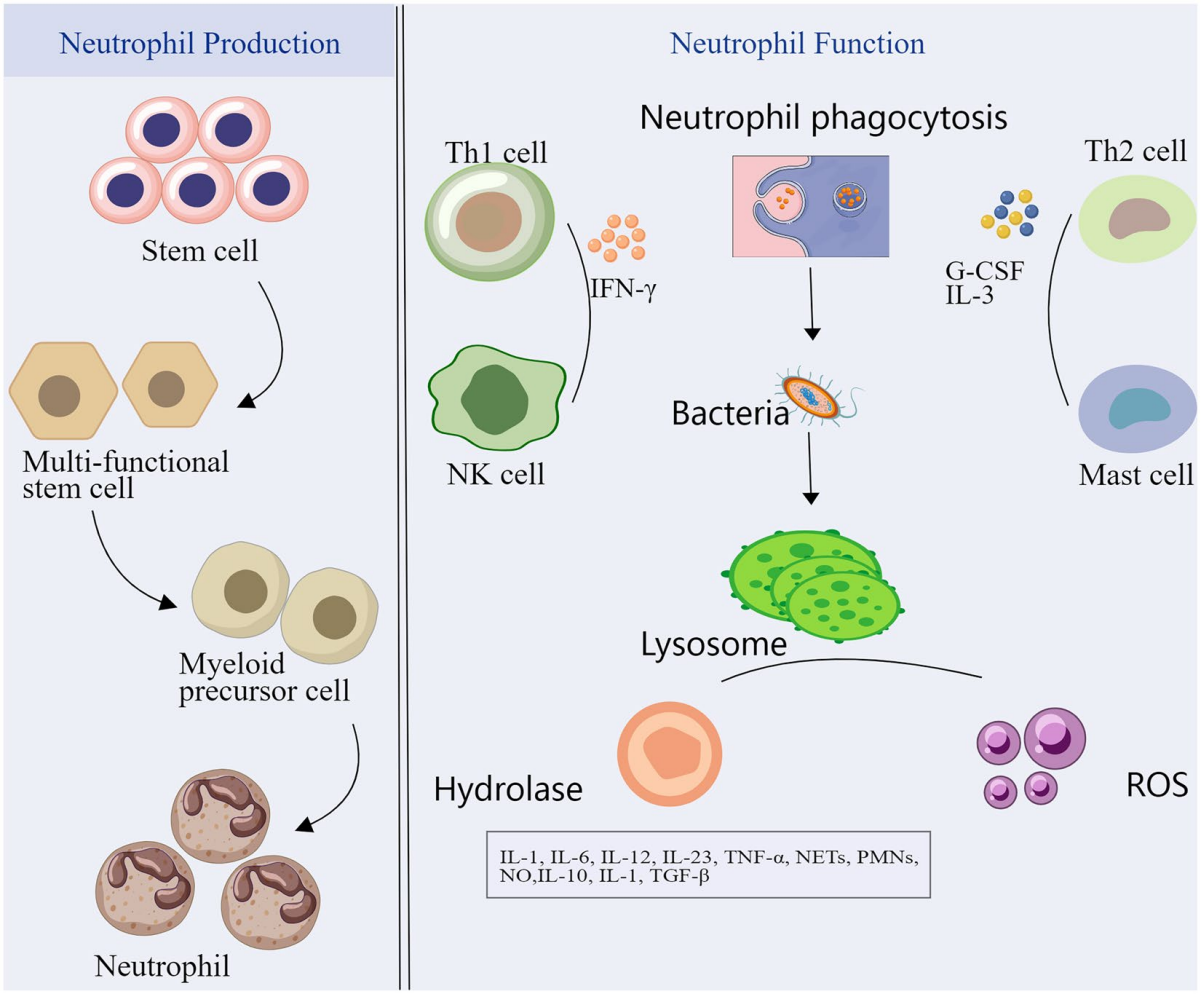
Production of neutrophils, recruitment of monocyte, function of neutrophils.

Neutrophils are a crucial type of white blood cell produced in the bone marrow, classified as polymorphonuclear leukocytes (PMNs) [6]. (Figure 1) They differentiate from hematopoietic stem cells in the bone marrow and undergo a series of developmental stages, including promyelocytes, late myelocytes, rod granulocytes, and ultimately mature into lobulated granulocytes. This maturation process is regulated by various cytokines and growth factors, such as granulocyte colony-stimulating factor (G-CSF) and interleukin-3 (IL-3) [7]. Once mature, neutrophils are released into the bloodstream, where they serve as the body’s first line of defense against infection. Although neutrophils have a relatively short lifespan in circulation, typically lasting only six to eight hours, they can survive for several days within tissues. They accumulate at sites of infection and inflammation, rapidly migrating to damaged tissues in response to chemokines, including interleukin-8 (IL-8) [8]. The production and release of neutrophils represent a vital response of the body to infection and inflammation. Infection or inflammatory stimuli can significantly enhance neutrophil production and mobilization through the release of cytokines and chemokines [9]. This phenomenon, known as neutrophilia, is characteristic of many infectious and inflammatory diseases.

Neutrophils play a crucial role in the body’s immune defense against pathogens through processes such as phagocytosis, degranulation, and the formation of extracellular traps. Phagocytosis is one of the most significant functions of neutrophils, enabling them to recognize and engulf bacteria, fungi, and other microorganisms by enclosing them in phagosomes [10]. These phagosomes subsequently fuse with lysosomes to form phagolysosomes, which release a variety of hydrolases and reactive oxygen species that are instrumental in killing and digesting pathogens. The degranulation of neutrophils involves the release of antimicrobial substances stored in intracellular granules. These granules include primary granules (myeloperoxidase, elastase), secondary granules (lactoferrin, lysozyme), and tertiary granules (gelatinase) [11,12]. These substances exhibit potent antibacterial activity, capable of directly killing pathogens or enhancing the functions of other immune cells. Additionally, neutrophils can trap and eliminate pathogens by forming NETs. NETs are mesh-like structures composed of deoxyribonucleic acid (DNA) and antibacterial proteins that entrap bacteria and fungi, thereby limiting their spread and facilitating their removal [13].

Neutrophils play a crucial role in the inflammatory response and are closely associated with a variety of inflammatory factors. They not only act as key mediators of the inflammatory response but are also significant sources of these factors. During infection or tissue injury, neutrophils facilitate the recruitment and activation of other immune cells by releasing cytokines and chemokines, thereby amplifying the inflammatory response [14]. The cytokines released by neutrophils include TNF, interleukin-1 (IL-1), and interleukin-6 (IL-6), among others. These factors are essential for regulating the inflammatory response, promoting increased vascular permeability, leukocyte infiltration, and acute phase protein synthesis [15]. Additionally, neutrophils release chemokines such as interleukin-8 (IL-8), which attract other neutrophils and monocytes to the site of inflammation. However, overactivation of neutrophils during the inflammatory response can result in tissue damage. The reactive oxygen species and proteases they release can effectively kill pathogens but may also harm host tissues, leading to uncontrolled and chronic inflammation [16]. In such instances, persistent inflammatory responses and tissue damage can contribute to the onset and progression of diseases such as chronic inflammation and airway damage in bronchiectasis.

## 2. Mechanism of action of neutrophils in bronchiectasis

### 2.1. Increased airway mucus secretion

Patients with bronchiectasis frequently exhibit excessive mucus secretion in the airways, resulting in airway obstruction and breathing difficulties. Neutrophils play a significant role in the production and secretion of mucus through various mechanisms. Inflammatory mediators, such as IL-8 and TNF, released by neutrophils, can stimulate airway epithelial cells and goblet cells to increase mucus secretion [17]. These mediators activate intracellular signaling pathways by binding to cell surface receptors, thereby enhancing the gene expression and secretion of mucin. Additionally, proteases released during neutrophil degranulation, including elastase and myeloperoxidase, can directly damage the airway epithelium, further promoting mucus secretion. The accumulation of neutrophils in the airways also impacts the properties of mucus through both physical and chemical mechanisms [18]. Neutrophils release DNA and NETs, which can increase the viscosity of mucus, making it more difficult to expel. This viscous mucus creates an optimal environment for pathogen proliferation, exacerbating infections and inflammatory responses. Moreover, neutrophils amplify the inflammatory response associated with mucus secretion by interacting with other immune cells [19]. The chemokines they release attract additional neutrophils and monocytes to the site of inflammation, establishing a continuous inflammatory response circuit [20]. This ongoing inflammatory stimulation results in a sustained increase in mucus secretion, which is a pathological hallmark of bronchiectasis.

### 2.2. Chronic inflammatory response

Patients with bronchiectasis frequently experience recurrent bacterial infections, which are closely associated with the ability of neutrophils to eliminate pathogens through mechanisms such as phagocytosis, the release of antimicrobial substances, and the formation of extracellular traps. The aggregation of neutrophils at the infection site is facilitated by various chemokines, including IL-8 and IL-1β [15,20]. These chemokines promote neutrophil migration by binding to specific receptors on their surface. Upon arrival at the infection site, neutrophils directly target pathogens by releasing reactive oxygen species and proteolytic enzymes. However, these substances can also inflict damage on host tissues, potentially resulting in uncontrolled and chronic inflammatory responses [21,22]. The role of neutrophils in chronic inflammation extends beyond direct pathogen elimination; they also play a crucial role in modulating the activity of other immune cells. Cytokines released by neutrophils can activate macrophages, lymphocytes, and other immune cells, thereby enhancing their capacity to combat infections [23,24]. Nevertheless, this amplified immune response may contribute to tissue damage and fibrosis in chronic inflammatory conditions, ultimately exacerbating bronchiectasis.

### 2.3. Airway injury and remodeling

A pathological feature of bronchiectasis is the structural destruction and remodeling of the airway wall, which is closely associated with the activation of neutrophils. Neutrophils directly impact airway structure by releasing various enzymes and active substances, resulting in damage and remodeling [25]. Proteases released from neutrophil granules, including elastase, collagenase, and matrix metalloproteinases (MMPs), degrade the extracellular matrix of the airway wall, leading to structural destruction [26]. These enzymes compromise the elasticity and strength of the airway wall by degrading elastin and collagen, resulting in dilation and deformation of the bronchi. Additionally, reactive oxygen species released by neutrophils can damage airway cell membranes and matrix proteins through oxidation, exacerbating airway structural destruction. The role of neutrophils in airway remodeling extends beyond structural damage; it also encompasses the regulation of proliferation and differentiation of airway wall cells. Cytokines released by neutrophils, such as transforming growth factor β (TGF-β) and platelet-derived growth factor (PDGF), stimulate the proliferation of fibroblasts and myofibroblasts, promoting fibrosis and thickening of the airway wall [27–29]. The combined effects of these active substances contribute to structural destruction and dysfunction of the airway. This pathological alteration not only impairs airway patency but also increases susceptibility to infection and inflammation, creating a vicious cycle.

### 2.4. Oxidative stress

During neutrophil activation, a significant amount of reactive oxygen species (ROS) is produced, which can cause oxidative damage to host tissues while effectively killing pathogens [30]. The ROS generated by neutrophils include superoxide anions, hydrogen peroxide, and hydroxyl radicals, which can lead to cell membrane damage, protein denaturation, and DNA breaks through the oxidation of lipids, proteins, and DNA [31]. Oxidative stress not only directly harms airway epithelial cells and matrix proteins but also triggers the production and release of inflammatory factors by activating signaling pathways, thereby amplifying inflammatory responses. Additionally, neutrophils play a role in regulating the function of the antioxidant system under oxidative stress conditions. The activities of the body’s antioxidant system, including superoxide dismutase (SOD), glutathione peroxidase (GPx), and catalase (CAT), may be inhibited, resulting in a reduced capacity to scavenge reactive oxygen species (ROS) [32,33]. This dysfunction of the antioxidant system further exacerbates oxidative stress and tissue damage. The impact of oxidative stress in bronchiectasis extends beyond tissue damage; it also influences the repair and remodeling processes of the airway. Oxidative stress can affect the proliferation, differentiation, and apoptosis of airway wall cells by regulating signaling pathways and gene expression, ultimately leading to remodeling and dysfunction of airway structure [34,35].

### 2.5. Immune system disorders

Patients with bronchiectasis frequently exhibit abnormal immune function, which is closely associated with the activation and dysfunction of neutrophils. The role of neutrophils in the immune system extends beyond their direct involvement in anti-infection responses; it also encompasses the regulation of other immune cells and the overall balance of immune responses [36]. Neutrophils contribute to immune system disorders primarily through increased chemotaxis and decreased apoptosis [37]. A hallmark pathological feature of bronchiectasis is the continuous aggregation and activation of neutrophils at the site of inflammation. Enhanced chemotaxis results in the accumulation of neutrophils at inflamed sites, exacerbating the inflammatory response and causing tissue damage. Additionally, reduced apoptosis of neutrophils impedes their clearance from these sites, creating a persistent inflammatory response circuit [38]. Neutrophils also influence the function of other immune cells; the cytokines and chemokines they release can modulate the activation and function of immune cells such as macrophages and lymphocytes, thereby disrupting the immune response. This dysregulation not only compromises the body’s ability to combat infections but may also contribute to the persistence of autoimmune responses and chronic inflammatory conditions.

## 3. The role of neutrophils in the early stages of bronchodilation

Neutrophils are among the most significant inflammatory cells involved in the early pathological processes of bronchiectasis, playing a crucial role in the initiation and progression of inflammation. Upon activation, neutrophils release a range of inflammatory mediators, including IL-8, TNF, IL-1β, as well as various chemokines and proteases. These mediators orchestrate inflammatory responses through intricate signaling networks, resulting in airway mucosal injury, structural destruction, and airway dysfunction [39,40].

Neutrophil elastase (NE) is a major protease in neutrophil primary granules, Bronchiectasis occurs early in the course of cystic fibrosis, is detectable in infants as young as 10 weeks of age, and is persistent and progressive. In a study of 127 infants with cystic fibrosis undergoing bronchoscopy, it was shown that NE activity in bronchoalveolar lavage fluid was a strong predictor of bronchiectasis risk. It is suggested that NE activity is associated with early bronchiectasis in children with cystic fibrosis [41]. Myeloperoxidase exacerbates airway inflammation and injury by promoting oxidative stress [42]. Recently, the role of NETs in bronchiectasis has garnered increasing attention. Research has shown that the sputum of patients with bronchiectasis contain substantial amounts of NETs, Sputum proteomics identified NET-associated proteins as being the most abundant in patients with bronchiectasis and the most closely associated with disease severity. In addition, sputum NET was associated with bronchiectasis severity index, quality of life, future hospitalization risk, and mortality [43]. Chang AB et al. [44] indicated that bronchiectasis in children may be partially reversible if diagnosed and treated at an early stage. Neutrophil-derived proteases are among the principal drivers of early airway remodeling in these patients; therefore, targeting neutrophilic inflammation during the ‘window of reversibility’ could halt or even reverse structural lung damage. Based on these studies, therapeutic strategies targeting neutrophil inflammatory mediators have become a new research focus.

## 4. The role of neutrophils in the Middle stage of bronchodilation

Neutrophils play a central role in sustaining the inflammatory response observed in mid-bronchiectasis. They contribute to the maintenance and exacerbation of airway inflammation through the release of numerous inflammatory mediators. Attracted to the site of inflammation by chemokines, neutrophils release reactive oxygen species, proteases, and cytokines. While these substances are effective in killing pathogens, they also inflict damage on host tissues. The reactive oxygen species released by neutrophils induce oxidative stress in the airway, which is a critical component of the inflammatory response. These species cause cellular damage and death by oxidizing lipids, proteins, and DNA. Furthermore, oxidative stress activates nuclear factor κB (NF-κB) and other signaling pathways, leading to increased expression and secretion of inflammatory factors, thereby establishing a positive feedback loop that perpetuates inflammation [45]. Studies have shown that blood reactive oxygen species (ROS) levels are significantly elevated in patients with bronchiectasis [46,47]. Reactive oxygen species can induce apoptosis and necrosis by oxidizing membrane lipids, proteins, and nucleic acids, resulting in the injury and shedding of airway epithelial cells. Furthermore, ROS not only directly damage cells and tissues but also promote the activation of matrix metalloproteinases through oxidative damage, which further contributes to tissue degradation and remodeling. During the middle stage of bronchodilation, neutrophils regulate the proliferation and migration of airway smooth muscle cells and fibroblasts by releasing cytokines and chemokines. Cytokines such as IL-8 and TNF bind to cell surface receptors, activating downstream signaling pathways that promote the proliferation and migration of airway smooth muscle cells, ultimately leading to airway stenosis and dysfunction [48]. Chemokines exacerbate airway inflammation by recruiting additional neutrophils and other inflammatory cells to the site of inflammation, thereby creating a positive feedback loop of inflammation.

Neutrophils play a crucial role in regulating mucus secretion in bronchiectasis, influencing airway mucus dynamics and clearance function. They modulate the secretory activity of goblet cells and mucus glands by releasing various cytokines and enzymes, which results in increased mucus secretion and alterations in its properties [49]. Excessive mucus secretion not only contributes to airway obstruction but also creates a favorable environment for pathogen proliferation, thereby exacerbating the inflammatory response. Additionally, neutrophils influence the activity and migration of other immune cells through the release of various cytokines and chemokines, thereby affecting the intensity and duration of the inflammatory response.

## 5. Effect of neutrophil reprogramming on bronchiectasis

A notable feature of neutrophil reprogramming is the increased secretion of chemotactic and inflammatory factors. Reprogrammed neutrophils tend to accumulate more readily in inflammatory areas and release a substantial quantity of pro-inflammatory factors locally, which not only exacerbates the local inflammatory response but may also induce systemic inflammation [50]. Concurrently, reprogrammed neutrophils exhibit heightened activity in the release of reactive oxygen species (ROS) and proteases, such as elastase, which can further damage airway epithelial cells and basement membranes, thereby promoting structural changes in the airway [51]. In addition to enhancing the immune response, neutrophil reprogramming may lead to an aberrant role in airway repair. Typically, damaged airway epithelial cells undergo normal repair mechanisms during recovery. However, reprogrammed neutrophils may interfere with airway recovery through inappropriate repair mechanisms and potentially contribute negatively to airway remodeling [52,53]. The continuous activation of these cells not only fails to effectively eliminate pathogens but may also result in fibrosis during airway repair, thereby worsening airway dilation and airflow restriction.

Recent studies have demonstrated that the function of neutrophils in the airways of patients with bronchiectasis exhibits a significant reprogramming characteristic. By analyzing airway secretions from these patients, researchers found that not only was there an increase in the number of neutrophils, but also a notable alteration in their cytokine secretion patterns. Compared to healthy individuals, neutrophils in bronchiectasis patients showed a significant increase in the secretion of pro-inflammatory cytokines, such as TNF-α and IL-8 [54]. This elevation in cytokine levels directly contributes to the enhancement of local inflammatory responses, thereby exacerbating airway injury and the airway remodeling process [55]. Furthermore, clinical studies have indicated that the reprogramming of neutrophils in bronchiectasis not only impacts the immune response within the airways but is also closely linked to the severity of clinical symptoms. Evidence suggests that the overactivation of neutrophils in these patients is strongly associated with persistent symptom exacerbation and a poor response to conventional therapies [56]. Consequently, targeting the reprogramming process of neutrophils may represent a novel therapeutic approach for bronchiectasis.

## 6. Treating bronchiectasis by targeting neutrophils

**Figure 2.**
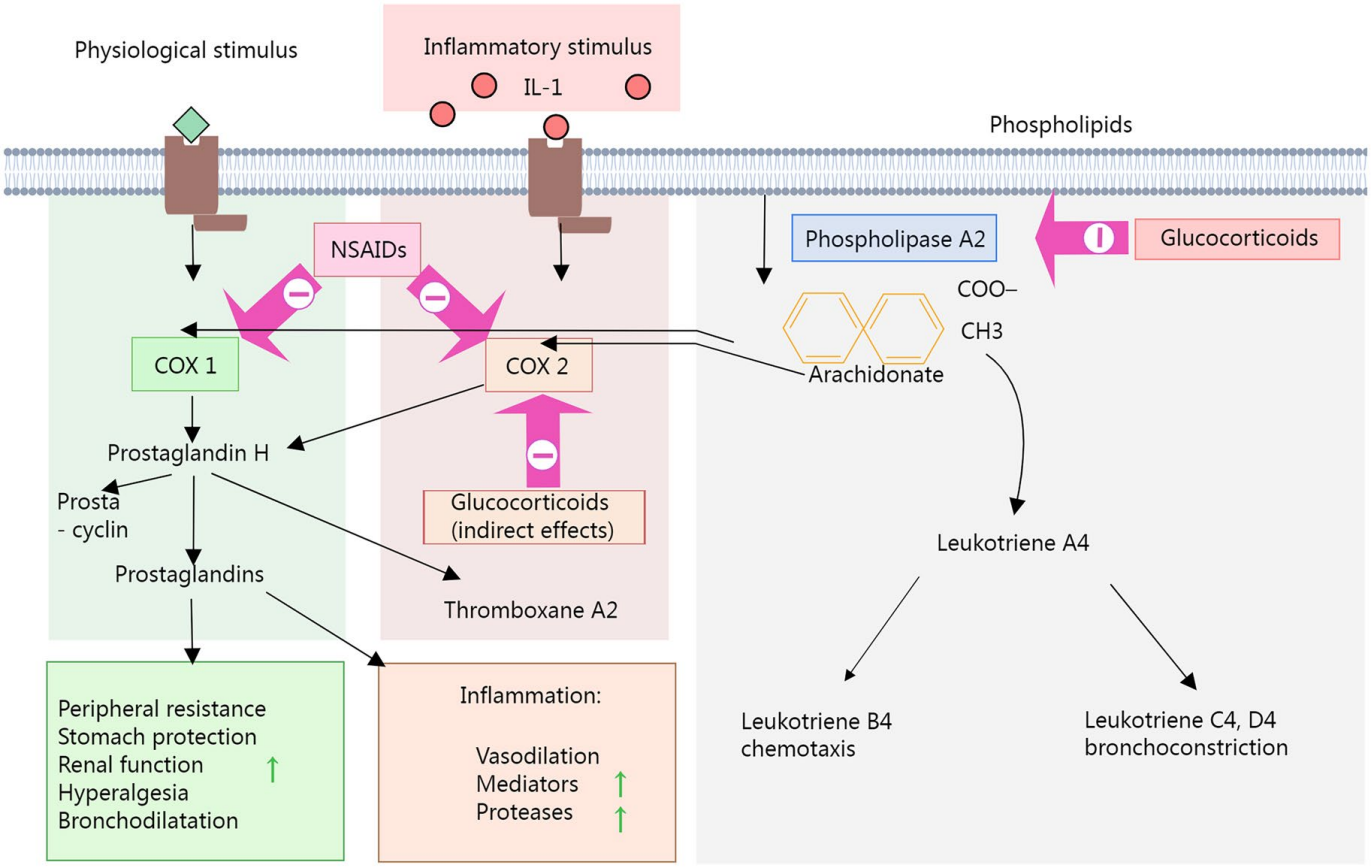
Mechanism of action of non-steroidal anti-inflammatory drugs (NSAIDs).

Glucocorticoids play a crucial role in the treatment of bronchiectasis by inhibiting the activation and migration of neutrophils, thereby reducing the release of inflammatory factors and mitigating the inflammatory response in the airways. Studies have demonstrated that glucocorticoids can significantly lower the levels of neutrophils and inflammatory factors in the sputum of patients with bronchiectasis, while also improving clinical symptoms and lung function [57]. Non-steroidal anti-inflammatory drugs (NSAIDs) alleviate inflammation by reducing the synthesis of prostaglandins through the inhibition of the cyclooxygenase (COX) enzyme [58]. (Figure 2) Although NSAIDs are effective in addressing acute inflammation, their role is limited in the context of chronic inflammation associated with bronchiectasis. The activation and persistence of neutrophils represent the core mechanism underlying chronic inflammation in this condition. Due to their relatively weak direct effect on neutrophils, NSAIDs are commonly utilized as adjuvant therapy in the treatment of bronchiectasis. Both glucocorticoids and NSAIDs work through distinct mechanisms to inhibit inflammation, reduce neutrophil activation and infiltration, and enhance patients’ clinical symptoms and quality of life. However, the long-term use of glucocorticoids may result in immunosuppression and other adverse effects, while NSAIDs can lead to gastrointestinal complications [59]. Therefore, it is essential to carefully weigh the efficacy and risks associated with these treatments in clinical practice and to formulate rational treatment plans.

Patients with bronchiectasis frequently experience chronic bacterial infections, with common pathogens including Pseudomonas aeruginosa and Haemophilus influenzae. The persistence of these bacteria significantly contributes to neutrophil activation and airway inflammation [60]. The primary objective of antibiotic therapy is to eliminate or control bacterial infections in the airway, thereby reducing the release of inflammatory mediators and minimizing neutrophil infiltration. Research has demonstrated that appropriate antibiotic treatment can notably decrease sputum volume and viscosity in patients with bronchiectasis, as well as enhance lung function and clinical symptoms [61]. Quinolones, cephalosporins, and macrolide antibiotics are commonly employed in the treatment of bronchiectasis. Among these, macrolide antibiotics not only exhibit antibacterial properties but also possess immunomodulatory and anti-inflammatory effects, which help to mitigate airway inflammation by inhibiting neutrophil chemotaxis and activation. However, the prolonged use of antibiotics may result in the emergence of drug-resistant strains and microbial dysbiosis. Therefore, the selection and administration of antibiotics in the treatment of bronchiectasis should be guided by the etiological findings and clinical manifestations of patients to formulate an individualized treatment plan.

ROS induce apoptosis and necrosis by oxidizing cell membrane lipids, proteins, and DNA, thereby compromising the structure and function of the airway. Antioxidants play a crucial role in preserving the integrity and function of airway epithelial cells by neutralizing reactive oxygen species and mitigating cellular damage associated with oxidative stress. Research has demonstrated that antioxidants can significantly lower oxidative stress levels in patients with bronchiectasis, reduce neutrophil infiltration and the release of inflammatory mediators, and enhance both the structure and function of the airway. Commonly utilized antioxidants include vitamin C, vitamin E, and NAC [62,63], among others. These compounds eliminate ROS through various mechanisms to alleviate cell damage resulting from oxidative stress. Notably, vitamin C is a water-soluble antioxidant that diminishes cellular damage by directly interacting with reactive oxygen species. Studies have demonstrated that vitamin C supplementation can significantly reduce oxidative stress levels in patients with bronchiectasis while improving airway structure and function [64]. Vitamin E, a fat-soluble antioxidant, protects membrane lipids from oxidative damage by integrating into cell membranes, thereby maintaining their integrity and functionality. Research indicates that vitamin E supplementation can diminish oxidative stress and neutrophil-mediated inflammatory responses in patients with bronchiectasis [65]. NAC, a precursor drug, enhances the antioxidant capacity of cells and mitigates cell damage induced by oxidative stress by increasing intracellular glutathione levels, Evidence suggests that NAC can significantly reduce oxidative stress and inflammatory responses in patients with bronchiectasis, leading to improvements in clinical symptoms and lung function [66]. Overall, antioxidants play a crucial therapeutic role in the management of bronchiectasis by eliminating reactive oxygen species, reducing oxidative stress-induced cellular damage, and protecting airway structure and function.

Neutrophils play a critical role in maintaining and exacerbating airway inflammation by releasing a diverse array of cytokines and chemokines during the chronic inflammation associated with bronchiectasis. Immunomodulators enhance airway structure and function by modulating the activity and function of immune cells, thereby influencing the intensity and duration of the inflammatory response. These agents help to regulate the balance and stability of the immune system through various mechanisms, reducing the activation and infiltration of neutrophils, which in turn alleviates chronic airway inflammation [67]. Commonly used immunomodulators encompass immunosuppressants, immune enhancers, and other immunomodulatory agents. These drugs operate through distinct mechanisms to restore balance and stability within the immune system, ultimately improving clinical symptoms and the quality of life for patients with bronchiectasis [68]. By inhibiting the activity and function of immune cells, immunosuppressants diminish neutrophil activation and infiltration, thereby mitigating chronic airway inflammation. Research has demonstrated that immunosuppressants can significantly lower levels of neutrophil infiltration and inflammatory mediators in patients with bronchiectasis, leading to enhancements in airway structure and function. Frequently utilized immunosuppressants include glucocorticoids, cyclosporin A, and tacrolimus, each of which inhibits immune cell activity through various mechanisms, further improving clinical symptoms and the quality of life for patients with bronchiectasis [69]. Immune enhancers augment the activity and functionality of immune cells, bolster the body’s ability to combat infections, diminish the activation and infiltration of neutrophils, and alleviate chronic airway inflammation. Research indicates that immune enhancers can significantly lower the levels of neutrophil infiltration and inflammatory factors in patients with bronchiectasis, while also enhancing airway structure and function [70]. Commonly utilized immune enhancers include interferon, interleukin-2, and thymosin. These agents enhance immune cell activity and functionality through various mechanisms, thereby improving clinical symptoms and quality of life for patients with bronchiectasis. Immunomodulins further contribute to the improvement of airway structure and function by modulating the activity and functionality of immune cells, which in turn influences the intensity and duration of inflammatory responses. Studies have demonstrated that immunomodulins can significantly reduce levels of neutrophil infiltration and inflammatory factors in bronchiectasis patients, leading to enhancements in airway structure and function. Frequently used immunomodulatory proteins include interleukin-10 and transforming growth factor-β, which regulate immune cell activity and functionality through distinct mechanisms, ultimately improving clinical symptoms in patients with bronchiectasis [71].

Recent advancements in the pharmacotherapeutic management of bronchiectasis have demonstrated significant clinical potential through the targeted inhibition of neutrophil-derived proteases, as evidenced by emerging clinical trial data. The ASPEN10 investigation, a comprehensive clinical study, has elucidated critical protocol parameters and baseline demographic characteristics associated with Brensocatib administration in non-cystic fibrosis bronchiectasis populations, establishing a foundation for its therapeutic application [72]. Preliminary evidence from the Phase 2 and Phase 3 randomized controlled trials evaluating the dipeptidyl peptidase-1 (DPP-1) inhibitor Brensocatib both revealed statistically significant reductions in pulmonary exacerbation frequency, indicating its potential efficacy in disease modification [73,74]. Mechanistic validation was further provided by the WILLOW89 study, which confirmed the compound’s capacity to potently inhibit neutrophil serine protease activity, including neutrophil elastase and proteinase 3, thereby attenuating the protease-antiprotease imbalance and subsequent neutrophilic airway inflammation and parenchymal destruction characteristic of bronchiectasis pathophysiology [75]. HSK31858 is currently being evaluated in a phase II trial for bronchiectasis, following a phase I study demonstrating its favorable safety profile and dose-dependent inhibition of NE activity [76]. BI 1291583, a selective cathepsin C (CatC) inhibitor, has shown dose-dependent CatC and NE suppression in phase I trials and demonstrated efficacy in the phase II AIRLEAF^®^ trial, significantly prolonging time to first exacerbation while maintaining a placebo-like safety profile, with further investigations ongoing in cystic fibrosis-related bronchiectasis and a planned phase III AIRTIVITY^®^ trial [77,78]. These cumulative findings substantiate the therapeutic rationale for neutrophil protease pathway modulation as a viable strategy in bronchiectasis management, with Brensocatib emerging as a promising therapeutic agent warranting further clinical evaluation.

Physical therapy and rehabilitation can enhance both the structure and function of the airway, decrease the activation and infiltration of neutrophils, and alleviate chronic inflammation within the airway. These interventions improve airway structure and function through various mechanisms, while also mitigating neutrophil activation and infiltration, and relieving chronic airway inflammation. Common methods employed in physical therapy and rehabilitation include breathing training, physical activity, and airway clearance techniques, each contributing to improvements in airway structure and function through distinct mechanisms. Breathing training, in particular, enhances the strength and endurance of respiratory muscles, increases lung capacity and airway patency, reduces neutrophil activation and infiltration, and alleviates chronic airway inflammation. Research indicates that breathing training can significantly enhance lung function in patients with bronchiectasis [79]. Physical activity reduces the activation and infiltration of neutrophils while alleviating chronic airway inflammation by enhancing the body’s endurance and anti-infection capabilities. Research indicates that appropriate levels of physical activity can significantly improve lung function and clinical symptoms in patients with bronchiectasis, thereby enhancing their quality of life [80]. Common forms of physical activity, such as walking, swimming, and cycling, contribute to the improvement of airway structure and function through various mechanisms. Additionally, airway clearance techniques minimize the activation and infiltration of neutrophils by eliminating airway secretions and pathogens, thus alleviating chronic airway inflammation. Studies have demonstrated that these airway clearing techniques can markedly enhance lung function and clinical symptoms in bronchiectasis patients, further improving their quality of life. Frequently utilized respiratory clearance methods include postural drainage, vibration sputum discharge, and airway cleaning devices. These technologies enhance airway structure and function through diverse mechanisms, ultimately improving clinical symptoms and the quality of life for patients with bronchiectasis [81].

## 7. Conclusion

As a crucial component of the immune system, the role of neutrophils in bronchiectasis has garnered significant attention from both clinicians and researchers. Although some studies have elucidated certain functions of neutrophils in this disease, many aspects of their specific pathogenesis remain unresolved. Currently, our understanding of how neutrophils mediate inflammatory responses at various stages of bronchiectasis, including the early and middle stages, is still insufficient. We anticipate that future research will illuminate the specific roles of neutrophils in bronchiectasis. Such studies will not only enhance our epidemiological data but will also promote high-quality clinical investigations into the multifaceted functions of neutrophils in this condition. Through these inquiries, we aim to gain a deeper understanding of how neutrophils influence disease progression and the presentation of patient symptoms. Furthermore, in-depth mechanistic studies will offer new insights into the treatment of bronchiectasis, particularly regarding precision medicine. By targeting specific neutrophil functions, it may be possible to develop effective treatment options that mitigate the disease’s impact on patients’ quality of life. Ultimately, these efforts will contribute to the achievement of individualized and precise management of bronchiectasis, thereby improving patient outcomes and quality of life.

## Data Availability

Data sharing is not applicable to this article as no data were created or analysed in this study.
